# Modelling of population structure through contemporary groups in genetic evaluation

**DOI:** 10.1186/s12863-019-0778-0

**Published:** 2019-10-24

**Authors:** Jaroslav Klápště, Mari Suontama, Heidi S Dungey, Emily J Telfer, Grahame T Stovold

**Affiliations:** 1 Scion (New Zealand Forest Research Institute Ltd.), 49 Sala Street, Rotorua, 3010 New Zealand; 2 Skogforsk, Umeå, Box 3, Sävar, 918 21 Sweden

**Keywords:** Linear mixed models, Genetic evaluation, Genetic groups, *Pseudotsuga menziesii*, Provenance/progeny test, Open-pollinated test

## Abstract

**Background:**

Forest trees can occupy extensive geography and environmentally highly variable areas which result in high genetic variability in the direction of pressure from natural selection. At the same time, the majority of conifer species are wind-pollinated from both short and long distances, resulting in wide-spread gene flow, which can lead to maladaptation to local conditions. Quantitative analyses of provenance/progeny tests correct for genetic differences between populations to ensure unbiased genetic parameters are obtained. Commonly, the provenance effect is fitted as a fixed term or can be implemented as a contemporary group in the pedigree.

**Results:**

The use of a provenance effect, either as a fixed term or as the same contemporary groups in both maternal and paternal sides of the pedigree, resulted in fairly similar precision of genetic parameters in our case. However, when we developed a phantom contemporary group for the paternal side of the pedigree that considered a different genetic quality of pollen compared with the maternal contribution from trees in the local environment, the model fit and accuracy of breeding values increased.

**Conclusion:**

Consideration of the mating dynamics and the vector of gene flow are important factors in modelling contemporary genetic groups, particularly when implementing pedigrees within a mixed model framework to obtain unbiased estimates of genetic parameters. This approach is especially important in traits involved in local adaptation.

## Background

Forest tree species usually occupy large geographical areas and are exposed to a wide range of environmental conditions. These large geographical ranges predetermine large genetic changes along the environmental gradients, such as latitude or altitude, through natural selection. The discovery of factors driving natural selection is essential for understanding a species’ adaptability and for the development of population management strategies suitable for changing climate conditions [1]. Common garden experiments, which include genetically broad material representing a large proportion of a species’ natural distribution, are a useful tool to dissect genetic divergence and phenotypic plasticity, both of which contribute to the phenotypic response to the changing environment [2]. The conclusions from such experiments are essential for effective implementation of seed transfer [3] or the extrapolation of population redistribution following predicted changes in climate conditions [4, 5]. In extreme cases, the results of climate change models show the change in the species distribution and thus local species composition [6].

Forest tree breeding populations are mostly in the initial stage of domestication, and thus the history of evolutionary processes involved in local adaptation such as migration, genetic drift, mutations and selection greatly affect the results of any initial genetic evaluations. The geographical differences in these processes should be included in these evaluations through the inclusion of a provenance effect, fitted either as a fixed or random effect [7]. Ugarte et al. [8] tested differences between models using genetic groups as fixed vs. random term in mixed linear models, and found an advantage from the later when males are assigned to genetic groups non-randomly to reduce prediction error variance. Since geographical structure in populations of forest tree species is present [9], the non-random contribution of males should be considered. Westell et al. [10] proposed using random genetic groups as a means to model the average effect of all phantom parents belonging to a particular genetic group in the evaluation of animal models, which is the favoured approach in genetic evaluations. Hadfield et al. [11] addressed several issues connected with using animal models in quantitative evolutionary genetics to infer natural selection in breeding values, namely their spatial structure and temporal changes. In particular, the assumption that the models must capture all factors contributing to the explanation of a phenotype’s variance to be considered close to the true model and obtain robust, unbiased estimates is often under question.

Douglas-fir (*Pseudotsuga menziesii* (Mirb.) Franco) is divided into two varieties: coastal Douglas-fir (*Pseudotsuga menziesii* var. *menziesii*) and interior Douglas-fir (*Pseudotsuga menziesii* var. *glauca*) [12]. A previous genetic marker-based analysis performed using allozymes found high population genetic differentiation (G _ST_∼ 0.24) in Douglas-fir compared with other conifer species [13], and 51% of that was attributed to the difference between the inland and coastal varieties [14]. Another Douglas-fir provenance study, including populations from British Columbia, Washington and Oregon, did not discover any strong patterns in productivity along geographical or climatic gradients. This could be explained by high sensitivity to microsite conditions, which was not considered in global patterns, or by the sampling strategy. However, the poorest performers on the maritime sites tested were originally from submaritime areas, indicating differences in ecotypic differentiation [3]. Earlier work by St Clair et al. [15] investigated provenance/progeny tests including provenances from Washington and Oregon, and constructed composite traits which strongly aligned to the east-west cline following temperature and elevation, and north-south cline following latitude and summer drought.

The aim of our study was to investigate the fitting of the provenance effect either as a fixed effect or as a contemporary group in the pedigree within a mixed model framework to reflect the best model (data) fit. In addition, the modelling of contemporary groups in the pedigree was included to reflect differences in the genetic composition of maternal and paternal contributions in species with long-distance pollen flow.

## Results

### Model fit and heritability

Four scenarios using a univariate mixed linear model were tested for each trait in each environment. Two basic models used provenance, as fixed (ABLUP-F) or random effect (ABLUP-R). Both had the poorest model fit in terms of Akaike’s Information Criterion (AIC). ABLUP-R mostly resulted in a better model fit compared to ABLUP-F with the exception of traits that showed statistically significant Q _ST_ produced by ABLUP-R model (Tables 1 and 2). Lastly, two models that used genetic groups implemented directly in the pedigree (ABLUP-GC1 and ABLUP-GC2) showed different model fit. While model ABLUP-GC2 showed similar or slightly improved AIC compared to ABLUP-F (Tables 1 and 3), ABLUP-GC1 showed the best model fit across all tested scenarios (Table 4). Similar to model fit, ABLUP-F and ABLUP-GC2 resulted in the identical heritability estimates ranging from 0.12 (MAL1) to 0.51 (VEL1) at Gowan Hill and from 0.04 (MAL1) to 0.84 (VEL1) at Kaingaroa. The model ABLUP-R resulted in slightly lower heritability estimates compared to ABLUP-F and reached values from 0.10 (BR2) to 0.47 (VEL1) at Gowan Hill and from 0.02 (MAL1) to 0.77 (VEL1) at Kaingaroa. The decrease in heritability estimates in ABLUP-R model was attributed to the fact that provenance effects were included in random terms and thus included in the denominator of heritability estimation. This model allowed for partitioning of genetic variance attributed to within provenance variance and within individuals between provenances and estimation of Q _ST_. The Q _ST_ values ranged from 0.032 (STR2) to 0.25 (DBH1) at Gowan Hill and from 0.00 (VEL1) to 0.27 (DBH1) at Kaingaroa (Table 2). The model ABLUP-GC2 resulted in heritability estimates similar to model ABLUP-F (Table 3). The best fit model (ABLUP-GC1) resulted in higher additive genetic as well as residual variance estimates, especially for traits showing high genetic differentiation (statistically significant Q _ST_). However, the level of heritability remained similar to other scenarios and reached values from 0.11 (BR2) to 0.46 (VEL1) at Gowan Hill and from 0.06 (MAL1 and AC2) to 0.74 (VEL1) at Kaingaroa (Table 4).

**Table 1:** Genetic parameters estimates using ABLUP-F model. Variance components, heritability (their standard errors in parentheses), breeding values accuracy and Akaike’s Information Criterion (AIC) for traits measured at Gowan Hill and Kaingaroa sites ABLUP-F model

Gowan Hill	Add.gen	Rep	Rep(Set)	Residual	*h* ^2^	r	AIC
DBH1	211 (34.3)	45.5 (13.6)	14.9 (4.76)	671 (31.0)	0.24 (0.04)	0.52	47691
STR1	0.12 (0.02)	0.02 (0.01)	0.01 (0.00)	0.24 (0.02)	0.29 (0.04)	0.54	737
MAL1	0.05 (0.01)	0.11 (0.03)	0.01 (0.00)	0.34 (0.01)	0.12 (0.03)	0.40	572
VEL1	0.06 (0.01)	0.01 (0.00)	0.00 (0.00)	0.05 (0.01)	0.51 (0.07)	0.54	-4242
DBH2	829 (133)	87.7 (28.0)	15.7 (13.8)	2305 (118)	0.27 (0.04)	0.53	52767
STR2	0.19 (0.02)	0.01 (0.00)	0.01 (0.00)	0.21 (0.02)	0.48 (0.06)	0.64	218
BR2	0.04 (0.02)	0.01 (0.00)	0.01 (0.00)	0.31 (0.01)	0.10 (0.03)	0.38	-135
MAL2	0.09 (0.02)	0.04 (0.01)	0.00 (0.00)	0.39 (0.02)	0.18 (0.04)	0.44	1391
AC2	0.49 (0.11)	0.10 (0.04)	0.03 (0.03)	1 (NA)	0.16 (0.03)	0.31	17094
Kaingaroa	Add.gen	Rep	Rep(Set)	Residual	*h* ^2^	r	AIC
DBH1	413 (62.5)	45.9 (14.5)	18.8 (6.69)	794 (52.9)	0.34 (0.05)	0.55	44636
STR1	0.06 (0.01)	0.06 (0.02)	0.01 (0.00)	0.35 (0.01)	0.14 (0.03)	0.40	792
MAL1	0.04 (0.04)	0.15 (0.04)	0.01 (0.01)	1.03 (0.05)	0.04 (0.04)	0.17	3208
VEL1	0.10 (0.03)	0.00 (0.00)	0.01 (0.01)	0.02 (0.02)	0.84 (0.21)	0.14	-586
DBH2	1050 (207)	78.0 (29.7)	30.1 (25.4)	2415 (183)	0.30 (0.06)	0.43	30872
STR2	0.10 (0.02)	0.08 (0.02)	0.01 (0.00)	0.34 (0.02)	0.23 (0.05)	0.38	747
BR2	0.04 (0.01)	0.00 (0.00)	0.01 (0.00)	0.21 (0.01)	0.15 (0.04)	0.33	-1076
MAL2	0.07 (0.04)	0.14 (0.04)	0.00 (0.00)	0.59 (0.05)	0.10 (0.07)	0.22	1176
AC2	0.20 (0.09)	0.09 (0.04)	0.06 (0.04)	1 (NA)	0.05 (0.02)	0.21	8373
NR2	0.04 (0.01)	0.19 (0.05)	0.02 (0.00)	0.23 (0.01)	0.14 (0.04)	0.32	-674

**Table 2:** Genetic parameters estimates using ABLUP-R model

Gowan Hill	Prov/Add.gen	Rep	Rep(Set)	Residual	*h*^2^/ *Q*_*ST*_	r	AIC
DBH1	140 (45.2) 212 (34.4)	45.3 (13.6)	14.9 (4.77)	671 (31.0)	0.21 (0.03) 0.25 (0.07)	0.52	47864
STR1	0.02 (0.01) 0.12 (0.02)	0.02 (0.01)	0.01 (0.00)	0.30 (0.02)	0.27 (0.04) 0.07 (0.03)	0.55	674
MAL1	0.01 (0.00) 0.05 (0.01)	0.11 (0.03)	0.01 (0.00)	0.34 (0.01)	0.11 (0.03) 0.08 (0.04)	0.41	495
VEL1	0.01 (0.00) 0.06 (0.01)	0.01 (0.00)	0.00 (0.00)	0.06 (0.01)	0.47 (0.06) 0.07 (0.03)	0.55	-4326
DBH2	412 (139) 835 (133)	88.3 (28.1)	15.3 (13.7)	2301 (118)	0.24 (0.04) 0.20 (0.06)	0.53	52972
STR2	0.01 (0.01) 0.18 (0.02)	0.01 (0.00)	0.01 (0.00)	0.21 (0.02)	0.45 (0.05) 0.03 (0.02)	0.65	153
BR2	0.00 (0.00) 0.03 (0.01)	0.01 (0.00)	0.01 (0.00)	0.31 (0.01)	0.10 (0.03) 0.05 (0.03)	0.38	-233
MAL2	0.02 (0.01) 0.09 (0.02)	0.04 (0.01)	0.00 (0.00)	0.39 (0.02)	0.17 (0.03) 0.10 (0.04)	0.44	1328
AC2	0.16 (0.07) 0.45 (0.10)	0.09 (0.04)	0.03 (0.03)	1 (NA)	0.14 (0.03) 0.15 (0.06)	0.31	16908
Kaingaroa	Prov/Add.gen	Rep	Rep(Set)	Residual	*h*^2^/ *Q*_*ST*_	r	AIC
DBH1	305 (90.1) 405 (61.4)	45.6 (14.5)	18.7 (6.69)	801 (52.2)	0.27 (0.04) 0.27 (0.04)	0.56	44840
STR1	0.00 (0.00) 0.05 (0.01)	0.06 (0.02)	0.01 (0.00)	0.35 (0.01)	0.13 (0.03) 0.03 (0.02)	0.41	694
MAL1	0.01 (0.01) 0.02 (0.04)	0.15 (0.04)	0.01 (0.01)	1.04 (0.04)	0.02 (0.03) 0.16 (0.24)	0.15	3142
VEL1	0.00 (0.00) 0.09 (0.02)	0.00 (0.00)	0.01 (0.01)	0.03 (0.02)	0.77 (0.18) 0.00 (0.00)	0.15	-637
DBH2	474 (156) 1027 (203)	77.4 (29.5)	30.5 (25.4)	2433 (181)	0.26 (0.05) 0.19 (0.06)	0.44	31092
STR2	0.01 (0.00) 0.09 (0.02)	0.08 (0.02)	0.01 (0.00)	0.34 (0.02)	0.20 (0.05) 0.04 (0.02)	0.39	666
BR2	0.00 (0.00) 0.03 (0.01)	0.00 (0.00)	0.01 (0.00)	0.22 (0.01)	0.13 (0.04) 0.04 (0.03)	0.34	-1177
MAL2	0.01 (0.01) 0.05 (0.04)	0.14 (0.04)	0.00 (0.00)	0.61 (0.04)	0.08 (0.06) 0.11 (0.10)	0.21	1116
AC2	0.08 (0.04) 0.16 (0.08)	0.09 (0.04)	0.06 (0.03)	1 (NA)	0.04 (0.02) 0.20 (0.12)	0.21	8263
NR2	0.01 (0.01) 0.03 (0.01)	0.19 (0.05)	0.02 (0.00)	0.23 (0.01)	0.12 (0.04) 0.17 (0.07)	0.32	-751

Variance components, heritability (their standard errors in parentheses), breeding values accuracy, Q _ST_ and Akaike’s Information Criterion (AIC) for traits measured at Gowan Hill and Kaingaroa sites ABLUP-R model

**Table 3:** Genetic parameters estimates using ABLUP-GC2 model

Gowan Hill	Add.gen	Rep	Rep(Set)	Residual	*h* ^2^	r	AIC
DBH1	211 (34.3)	45.5 (13.6)	14.9 (4.76)	671 (31.0)	0.24 (0.04)	0.53	47691
STR1	0.12 (0.02)	0.02 (0.01)	0.01 (0.00)	0.29 (0.02)	0.29 (0.04)	0.56	737
MAL1	0.05 (0.01)	0.11 (0.03)	0.01 (0.00)	0.34 (0.01)	0.12 (0.03)	0.30	572
VEL1	0.06 (0.01)	0.01 (0.00)	0.00 (0.00)	0.05 (0.01)	0.51 (0.07)	0.56	-4242
DBH2	829 (133)	87.7 (28.0)	15.7 (13.8)	2305 (118)	0.27 (0.04)	0.54	52767
STR2	0.19 (0.02)	0.01 (0.00)	0.01 (0.00)	0.21 (0.02)	0.48 (0.06)	0.66	218
BR2	0.04 (0.01)	0.01 (0.00)	0.01 (0.00)	0.31 (0.01)	0.10 (0.03)	0.37	-135
MAL2	0.09 (0.02)	0.04 (0.01)	0.00 (0.00)	0.39 (0.02)	0.18 (0.04)	0.44	1391
AC2	0.57 (0.13)	0.10 (0.04)	0.03 (0.03)	1 (NA)	0.19 (0.03)	0.49	17129
Kaingaroa	Add.gen	Rep	Rep(Set)	Residual	*h* ^2^	r	AIC
DBH1	419 (63.0)	45.9 (14.6)	18.7 (6.69)	790 (53.3)	0.35 (0.05)	0.58	44644
STR1	0.06 (0.01)	0.06 (0.02)	0.01 (0.00)	0.35 (0.01)	0.14 (0.03)	0.36	788
MAL1	0.04 (0.04)	0.15 (0.04)	0.01 (0.01)	1.03 (0.05)	0.04 (0.04)	NA	3207
VEL1	0.10 (0.03)	0.00 (0.00)	0.01 (0.01)	0.02 (0.02)	0.84 (0.21)	0.25	-586
DBH2	1050 (207)	78.4 (29.8)	30.3 (25.4)	2416 (184)	0.30 (0.06)	0.46	30879
STR2	0.10 (0.02)	0.08 (0.02)	0.01 (0.00)	0.34 (0.02)	0.22 (0.05)	0.37	743
BR2	0.04 (0.01)	0.00 (0.00)	0.01 (0.00)	0.21 (0.01)	0.14 (0.04)	0.33	-1080
MAL2	0.07 (0.04)	0.14 (0.04)	0.00 (0.00)	0.60 (0.05)	0.09 (0.07)	0.20	1173
AC2	0.20 (0.09)	0.09 (0.04)	0.06 (0.04)	1 (NA)	0.05 (0.02)	0.19	8369
NR2	0.04 (0.01)	0.19 (0.05)	0.02 (0.00)	0.23 (0.01)	0.13 (0.04)	0.17	-678

Variance components, heritability (their standard errors in parentheses), breeding values accuracy and Akaike’s Information Criterion (AIC) for traits measured at Gowan Hill and Kaingaroa sites ABLUP-GC2 model

**Table 4:** Genetic parameters estimates using ABLUP-GC1 model

Gowan Hill	Add.gen	Rep	Rep(Set)	Residual	*h* ^2^	r	AIC
DBH1	217 (35.1)	45.5 (13.6)	14.9 (4.76)	721.2 (24.2)	0.23 (0.03)	0.68	47602
STR1	0.12 (0.02)	0.02 (0.01)	0.01 (0.00)	0.32 (0.01)	0.27 (0.04)	0.70	663
MAL1	0.05 (0.01)	0.11 (0.03)	0.01 (0.00)	0.35 (0.03)	0.12 (0.03)	0.55	500
VEL1	0.06 (0.01)	0.01 (0.00)	0.00 (0.00)	0.07 (0.01)	0.46 (0.05)	0.71	-4314
DBH2	851 (136)	87.7 (28.0)	15.8 (13.8)	2501 (91.4)	0.25 (0.04)	0.69	52676
STR2	0.19 (0.02)	0.01 (0.00)	0.01 (0.00)	0.25 (0.01)	0.43 (0.05)	0.75	143
BR2	0.04 (0.01)	0.01 (0.00)	0.01 (0.00)	0.32 (0.01)	0.11 (0.03)	0.59	-206
MAL2	0.09 (0.02)	0.04 (0.01)	0.00 (0.00)	0.41 (0.01)	0.18 (0.03)	0.63	1318
AC2	0.51 (0.11)	0.10 (0.04)	0.03 (0.03)	1 (NA)	0.17 (0.03)	0.32	17006
Kaingaroa	Add.gen	Rep	Rep(Set)	Residual	*h* ^2^	r	AIC
DBH1	428 (64.4)	45.9 (14.6)	18.7 (6.69)	889 (40.0)	0.33 (0.04)	0.71	44560
STR1	0.06 (0.01)	0.06 (0.02)	0.01 (0.00)	0.36 (0.01)	0.14 (0.03)	0.60	714
MAL1	0.06 (0.04)	0.15 (0.04)	0.01 (0.01)	1.02 (0.04)	0.06 (0.04)	0.35	3135
VEL1	0.11 (0.03)	0.00 (0.00)	0.01 (0.01)	0.04 (0.02)	0.74 (0.14)	0.54	-640
DBH2	1085 (212)	78.3 (29.8)	30.5 (25.4)	2659 (141)	0.29 (0.05)	0.65	30794
STR2	0.10 (0.02)	0.08 (0.02)	0.01 (0.00)	0.36 (0.02)	0.22 (0.05)	0.59	668
BR2	0.04 (0.01)	0.00 (0.00)	0.01 (0.00)	0.22 (0.01)	0.15 (0.04)	0.57	-1154
MAL2	0.09 (0.05)	0.14 (0.04)	0.00 (0.00)	0.60 (0.04)	0.13 (0.07)	0.43	1100
AC2	0.23 (0.09)	0.09 (0.04)	0.05 (0.04)	1 (NA)	0.06 (0.02)	0.24	8299
NR2	0.04 (0.01)	0.19 (0.05)	0.02 (0.00)	0.24 (0.01)	0.15 (0.04)	0.41	-750

Variance components, heritability (their standard errors in parentheses), breeding values accuracy and Akaike’s Information Criterion (AIC) for traits measured at Gowan Hill and Kaingaroa sites ABLUP-GC1 model

### Accuracy of breeding value estimates

**Fig. 1:**
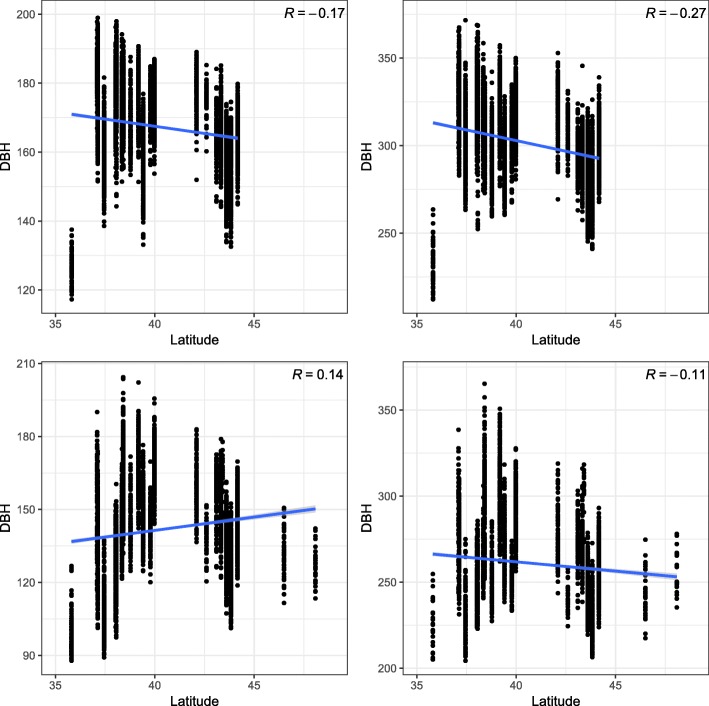
Distribution of breeding values estimated for DBH along latitude of origin. Latitudinal distribution of individual estimated breeding values (EBV) for DBH at Gowan Hill at age of 11 (upper left) and at age of 21 (upper right). At Kaingaroa, the individual estimated breeding values (EBV) at the age of 11 (bottom left) and at age of 21 (bottom right)

**Fig. 2:**
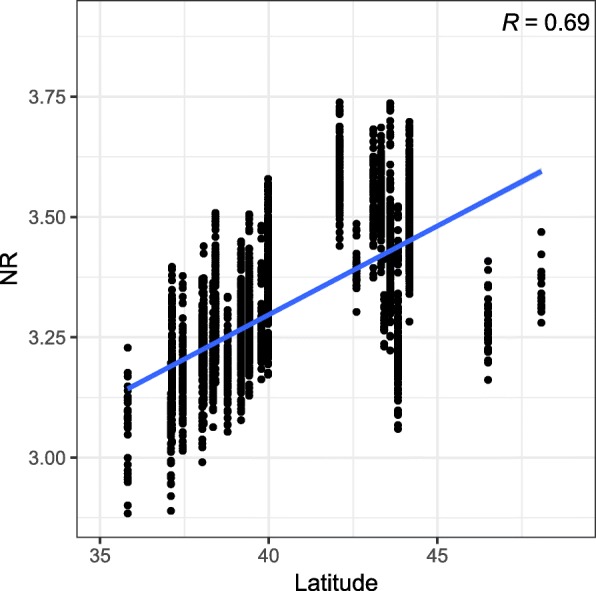
Distribution of breeding values estimated for NR along latitude of origin. Latitudinal distribution of individual breeding values (EBV) for needle retention at Kaingaroa at the age of 21

The models ABLUP-F and ABLUP-R resulted in similar accuracy of breeding value estimates reaching values from 0.31 (AC2) to 0.65 (STR2) at Gowan Hill and from 0.14 (VEL1) to 0.56 (DBH1) at Kaingaroa (Tables 1 and 2). The model ABLUP-GC2 resulted in the lowest accuracy of breeding value estimates which ranged from 0.30 (MAL1) to 0.66 (STR2) at Gowan Hill and from 0.17 (NR2) to 0.58 (DBH1) at Kaingaroa (Table 3). The best fit model (ABLUP-GC1), resulted in the highest accuracy of breeding value estimates ranging from 0.32 (AC2) to 0.75 (STR2) at Gowan Hill and from 0.24 (AC2) to 0.71 (DBH1) at Kaingaroa (Table 4). The improvement in breeding values accuracy was noticeable, especially in traits with statistically significant Q _ST_ (DBH and NR). The accuracy of breeding values for these traits was lowest in the ABLUP-F model, where values ranged from 0.32 to 0.55. Similar accuracy was reached in ABLUP-CG2, with values ranging from 0.17 to 0.58. When the ABLUP-CG1 model was implemented, the accuracy of breeding values increased, and values ranged from 0.41 to 0.71. For example, the accuracy of breeding values for DBH1 at Gowan Hill increased from 0.53 (ABLUP-GC2) to 0.68 (ABLUP-GC1). Similarly, the accuracy of breeding values for NR2 at Kaingaroa increased from 0.17 (ABLUP-GC2) to 0.41 (ABLUP-GC1) (Tables 1, 2, 3 and 4). The correlation of latitude of origin with breeding values estimated for DBH found that productivity increased with decreasing latitude (Fig. 1 - upper row) which was more obvious at later age (correlation of -0.17 versus -0.27). The opposite pattern was observed at Kaingaroa at an early age and was reversed at a later age (correlation of 0.14 versus -0.11) (Fig. 1 - bottom row). This pattern observed at Kaingaroa resulted from the presence of a needle disease termed Swiss needle cast (SNC), caused by *Phaeocryptopus gaeumannii* [16]. The needle retention trait, indirectly inferring resistance to disease, was therefore scored, evaluated and showed pattern found for productivity at an early age (Fig. 2). However, opposite pattern in productivity at a later age (Fig. 1 - bottom right) would assume that decreased productivity of most sensitive provenances at an early age (Fig. 1 - bottom left) is diminished with age.

### Genetic correlations

**Fig. 3:**
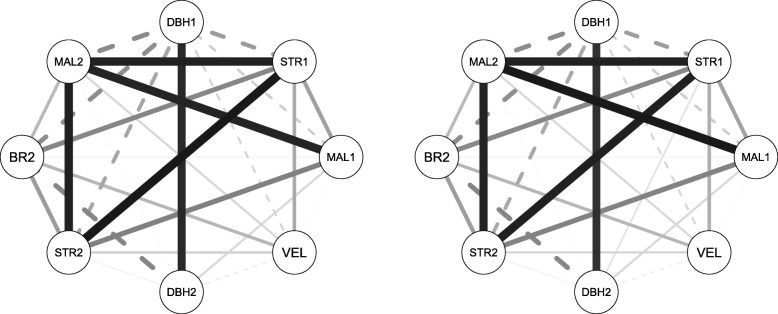
Network of trait’s genetic correlations at Gowan Hill. Correlation network estimated by the model using provenances as a fixed term (ABLUP-F) (left) or implemented as genetic groups in the pedigree (ABLUP-CG1) (right) at Gowan Hill

**Fig. 4:**
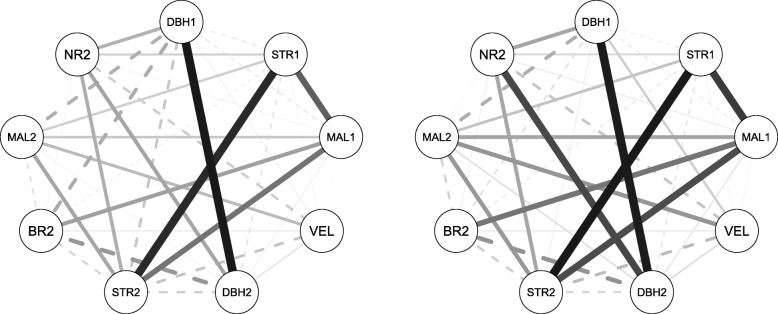
Network of trait’s genetic correlations at Kaingaroa. Correlation network estimated by the model using provenances as a fixed term (ABLUP-F) (left) or implemented as genetic groups in the pedigree (ABLUP-CG1) (right) at Kaingaroa

A bivariate mixed linear model was implemented for the estimation of pair-wise genetic correlations using ABLUP-F (the default model) and ABLUP-CG1 (the model which showed best AIC for all traits). Both models resulted in similar genetic correlations with slightly lower estimates in the ABLUP-CG1 model. The results show a negative genetic correlation of DBH1 to all other tested traits using both models, with the exception of positive genetic correlations to DBH2 and NR2 at Kaingaroa. Both STR1 and STR2 traits showed strong positive genetic correlations to other stem form traits such as MAL1, MAL2, and BR2, ranging from 0.34 to 0.93 at Gowan Hill and from -0.09 to 0.97 at Kaingaroa. BR2 is the only trait that showed an opposite pattern between sites. While strong positive genetic correlations between BR2 and form traits such as STR1, MAL1 and STR2, ranging from 0.06 to 0.50, were observed at Gowan Hill, only moderate correlations ranging from -0.21 to 0.58, were observed at Kaingaroa. VEL1 did not show any statistically significant genetic correlations to any other traits, probably due to low sample size (only 61% of individuals were measured at Gowan Hill, and 10% of individuals were measured at Kaingaroa for this trait). The traits measured at both ages (DBH, STR, MAL) showed strong age x age correlations, ranging from 0.86 to 0.96 at Gowan Hill and from 0.23 to 0.97 at Kaingaroa, indicating the stability of these traits’ expression levels across the investigated developmental stages (Tables 5 and 6; Figs. 3 and 4). The exploration of genotype by environment interaction (GxE) was performed through genetic correlations between environments. While STR and VEL reached high genetic correlations, indicating no GxE, other traits showed lower correlations (under 0.7) indicating the presence of strong GxE. DBH, measured at both ages, showed an increase in GxE with increasing age (Table 7). The Mantel test performed on the correlation matrices derived from ABLUP-F and ABLUP-CG1 models resulted in correlations of 0.99 in Gowan Hill and 0.97 in Kaingaroa. The modularity test showed that the majority of traits are independent, with the exceptions of MAL and STR in Gowan Hill belonging to a single module in both of the tested models. Additionally, DBH2 and NR2 were detected as traits belonging to the same module in Kaingaroa but only in the ABLUP-CG1 model.

**Table 5:** Genetic correlation estimates at Gowan Hill

Gowan Hill	DBH1	STR1	MAL1	VEL1	DBH2	STR2	BR2	MAL2
DBH1	1	-0.41 (0.10)	-0.21 (0.14)	-0.17 (0.11)	0.86 (0.04)	-0.36 (0.09)	-0.48 (0.13)	-0.48 (0.11)
STR1	-0.41 (0.10)	1	0.39 (0.12)	0.34 (0.10)	0.12 (0.05)	0.93 (0.03)	0.50 (0.13)	0.93 (0.07)
MAL1	-0.20 (0.14)	0.38 (0.12)	1	0.02 (0.13)	0.17 (0.14)	0.53 (0.11)	0.06 (0.17)	0.96 (0.10)
VEL1	-0.17 (0.11)	0.34 (0.10)	0.02 (0.13)	1	-0.08 (0.11)	0.18 (0.10)	0.30 (0.13)	0.17 (0.12)
DBH2	0.86 (0.04)	0.00 (0.11)	0.16 (0.13)	-0.08 (0.11)	1	0.05 (0.10)	-0.51 (0.13)	-0.04 (0.13)
STR2	-0.36 (0.09)	0.92 (0.03)	0.51 (0.10)	0.18 (0.10)	0.05 (0.10)	1	0.41 (0.12)	0.92 (0.05)
BR2	-0.45 (0.13)	0.48 (0.12)	0.06 (0.17)	0.29 (0.13)	-0.49 (0.13)	0.40 (0.12)	1	0.29 (0.15)
MAL2	-0.47 (0.11)	0.89 (0.07)	0.88 (0.10)	0.16 (0.12)	-0.04 (0.13)	0.90 (0.05)	0.28 (0.15)	1

Genetic correlation between traits (their standard errors in parentheses) measured at Gowan Hill, estimated using ABLUP-GC1 model (above diagonal) and ABLUP-F model (below diagonal)

**Table 6:** Genetic correlation estimates at Kaingaroa

Kaingaroa	DBH1	STR1	MAL1	VEL1	DBH2	STR2	BR2	MAL2	NR2
DBH1	1	-0.07 (0.13)	-0.08 (0.28)	0.20 (0.23)	0.96 (0.01)	0.05 (0.07)	-0.13 (0.09)	-0.34 (0.23)	0.38 (0.14)
STR1	-0.09 (0.13)	1	0.83 (0.28)	0.03 (0.31)	-0.11 (0.15)	0.97 (0.09)	-0.09 (0.19)	0.26 (0.27)	0.16 (0.20)
MAL1	-0.09 (0.23)	0.67 (0.22)	1	0.03 (0.59)	0.10 (0.33)	0.75 (0.34)	0.58 (0.43)	0.39 (0.55)	0.07 (0.41)
VEL1	0.05 (0.24)	0.03 (0.31)	0.01 (0.48)	1	0.13 (0.24)	-0.29 (0.27)	0.09 (0.28)	0.44 (0.46)	-0.25 (0.28)
DBH2	0.96 (0.02)	-0.11 (0.15)	0.05 (0.26)	0.11 (0.24)	1	-0.18 (0.15)	-0.46 (0.16)	0.12 (0.14)	0.78 (0.06)
STR2	-0.24 (0.13)	0.89 (0.09)	0.58 (0.26)	-0.25 (0.27)	-0.20 (0.15)	1	-0.21 (0.19)	0.43 (0.25)	0.38 (0.19)
BR2	-0.35 (0.15)	-0.06 (0.19)	0.40 (0.32)	0.08 (0.28)	-0.43 (0.16)	-0.17 (0.19)	1	-0.19 (0.32)	0.06 (0.22)
MAL2	-0.33 (0.20)	0.21 (0.25)	0.23 (0.41)	0.28 (0.41)	0.04 (0.23)	0.37 (0.23)	-0.13 (0.28)	1	0.02 (0.34)
NR2	0.32 (0.14)	0.19 (0.18)	0.04 (0.32)	-0.21 (0.27)	0.38 (0.15)	0.34 (0.18)	0.02 (0.21)	0.01 (0.29)	1

Genetic correlation between traits (their standard errors in parentheses) measured at Kaingaroa, estimated using ABLUP-GC1 model (above diagonal) and ABLUP-F model (below diagonal)

**Table 7:** Genetic correlation between sites

Trait	ABLUP-F	ABLUP-GC1
DBH1	0.68 (0.08)	0.66 (0.08)
STR1	0.87 (0.11)	0.81 (0.10)
MAL1	0.77 (0.46)	0.52 (0.30)
VEL1	0.76 (0.14)	0.70 (0.15)
DBH2	0.43 (0.12)	0.42 (0.12)
STR2	0.85 (0.09)	0.81 (0.09)
BR2	0.69 (0.19)	0.61 (0.18)
MAL2	0.53 (0.25)	0.46 (0.23)

Genetic correlations between sites using ABLUP-F model and ABLUP-CG1 model

## Discussion

### Population structure and its relevance in forest trees

Local adaptation is the result of evolutionary forces such as migration, random drift, natural selection and mutation. Contemporary groups can efficiently model genetic differences between sets of individuals coming from different environments undergoing different directions of local adaptation. However, the mobility of seed and pollen in wind-pollinated species such as conifers is different [17], and both parents do not necessarily come from the same genetic group. While seed dispersion is realised mostly within 60 meters, pollen dispersal is usually within the range of several hundreds of metres [18], however, this can reach as much as 500 - 750 km in conifer species [19, 20]. Moreover, while a seed donor is considered adapted to the local environment due to survival and successfully reaching sexual maturity (see Box 2 in [21] for reference), the pollen donor has not necessarily interacted with the local environment due to the possibility of long-distance pollen transfer.

The investigation of evolutionary responses across populations sampled along different environmental gradients and planted under a common environment is critical for understanding population genetic divergence and will guide the selection of material suitable for future climatic conditions [2]. However, using appropriately informed models is necessary to obtain an unbiased estimation of genetic parameters and make correct inferences about evolutionary responses [11], which is especially important in traits responsible for local adaptation. Most of the conifer domestication programmes are in their initial phases, where samples are collected across a wide range of environments and planted under common environmental conditions [22, 23]. Under such conditions, population genetic divergence provides important information about the strength of local adaptation and its modelling is critical to obtain accurate genetic parameters about the evolutionary capacity to respond to future climate conditions [11].

The current intensity of climate change is placing pressure on forest tree populations to improve resilience to increasingly variable environments during their long lifespans. Widely distributed conifer species are able to promote local adaptation due to intensive gene flow. However, it might not be enough to cope with rapid climate change [24]. Climate and growth models have estimated that maladaptation to future climate results not only in reduced productivity and increased mortality [25] but also in increased sensitivity to pathogens [26]. Therefore, active matching of forest populations to future climate conditions [4, 5, 24, 27], as well the monitoring and assessing host-pathogen interaction is required for optimal resilience [28]. In extreme cases, this type of active management can lead to a change in species composition [6].

### Modelling of population structure in genetic evaluation

The population structure is usually fitted as either fixed or random term in the genetic evaluation using mixed linear models [8]. The modelling of population structure through contemporary groups implemented directly in pedigree improved model fit and increased the accuracy of breeding values in the current study. However, assigning both maternal and paternal contribution to the same contemporary group resulted in the same model fit as using provenance as a fixed term. Consequently, defining only a different contemporary group for the paternal side of the pedigree, reflecting a different genetic background, resulted in improvement of both model fit and the accuracy of breeding values. In addition, models considering provenance as a fixed term or including both parents in the same contemporary group resulted in slightly higher heritability, than different contemporary groups in the both sides of the pedigree. These results indicate that the incorrect fit of the population structure would result in a somewhat overestimated genetic parameters. The different genetic groups for maternal and paternal contributions is expected, this is supported by the research results of mating dynamics in seed orchards. Evidence was shown on the presence of pollen contamination in seed crop in the range of 10% [29] to 90% [30] with indicating a relatively stable seasonal variability [29, 31].

The use of genetic groups provides flexibility in modelling of population structure, which corresponds to real-life seed and pollen mobility [18–20] and should be preferred over the fitting of provenance as a fixed term. However, in the case of insect-pollinated species, pollen mobility is directed by the pollinator and is usually limited only to the local population. Gonzaga et al. [32] investigated mating patterns in Eucalyptus seed orchard and found average pollen dispersal of 94 metres and pollen contamination of 14%.

Similarly, Rao et al. [33] reported mean pollen contamination of 17.6% in Eucalyptus breeding arboretum. Under such conditions, modelling of both the maternal and paternal sides of pedigree by the same contemporary group is a sensible solution, due to the fact that both parents survived and reached sexual maturity under the same environmental conditions and thus passed the same kind of natural selection (see Box 2 in [21] for reference). The development of genetic markers through next-generation sequencing platforms such as "Genotyping-by-sequencing" [34] can help to improve the inference about population structure. Although, a thorough understanding of gene flow on a landscape scale is anyhow challenging even with the help of genetic markers [35].

### Benefits of modelling of population structure for traits potentially involved in local adaptation

Our study investigated investigated the level of genetic differentiation between populations for all investigated traits through Q _ST_ parameters and found them as statistically significant based on their standard errors for productivity traits (DBH) and foliar disease resistance (to SNC) measured by needle retention (NR2). Similar levels of between-population differentiation were found for other potentially adaptive traits in conifers such as bud set, bud flush and cold injury [36]. Previous studies of genetic differentiation in coastal Douglas-fir found very low F _ST_ values ranging between 0.006 and 0.071 [14, 37, 38]. Additionally, Wilhelmi et al. [39] found differences in needle retention between provenances and concluded that provenances originating from areas of higher foliar disease pressure are more resistant. It can be therefore assumed that these traits are contributing to local adaptation. The considerable genetic variability between populations (large Q _ST_) indicates a high level of local natural selection and thus adaptive potential in traits related to productivity [40]. The exploration of provenance performance along latitudes showed that the most productive provenances tested in New Zealand originate from latitude around 38^∘^ N at Gowan Hill and around 40^∘^ N at Kaingaroa (Fig. 1). The advantage of more northern provenances at Kaingaroa (Latitude 38^∘^ 17’ S) can be attributed to the reduced needle retention of the southern provenances (Fig. 2) due to poorer resistance to SNC [16]. Douglas-fir at Kaingaroa is exposed to SNC due to the favourable climate for the disease in the North Island, such as mild mean daily winter temperatures and high spring moisture [41]. Therefore, it is critical to include needle retention as selection criteria in the North Island of New Zealand to improve resistance to SNC in Douglas-fir [42].

Correlation analysis discovered strong positive genetic correlations between NR2 and DBH at Kaingaroa. This relationship was more pronounced at later age, which indicates that reduced productivity depends on the first occurrence and frequency of foliar diseases. The correlations were mostly similar between implemented methods. However, some correlation estimates were stronger at Kaingaroa when contemporary groups were implemented in the pedigree compared with a model using provenance as a fixed term. Nevertheless, the Mantel test found high agreement between correlation matrices from tested models, reaching 0.99 in Gowan Hill and 0.97 in Kaingaroa. Appropriate modelling of population structure is critical to obtain reliable estimates of genetic correlations which can be implemented in evolutionary response to selection [11]. Additionally, reliable estimates of genetic correlations are required in the evolutionary developmental biology field of research to infer organismal modularity [43] and phenotypic integration [44]. Our test of modularity identified a module consisting of productivity (DBH2) and disease resistance (NR2) traits, both identified as traits potentially involved in the process of local adaptation in environment where both traits were expressed at the Kaingaroa site. This represents traits varying in the same way independently from other traits [45]. Interestingly, this module was identified only in the model that used different contemporary groups in the maternal and paternal sides of the pedigree. Therefore, the appropriate modelling of population structure is especially critical for traits involved in local adaptation showing a decent level of genetic divergence at the population level - Q _ST_). However, it is worth to mention that the resulting effect of population structure modelling depends on the extent of genetic variation included in the tested sample. Ideally, it should include a sample representing the whole natural distribution of the species. In our case, the sample represented populations from only part of the natural distribution (Oregon and California).

In contrast to the testing of provenance effect as a fixed term in mixed models, the implementation of contemporary groups in the pedigree allows greater flexibility in the modelling of differences in evolutionary forces, such as migration and natural selection between seed and pollen. This is important, especially in wind-pollinated species showing long-distance gene flow across heterogeneous environments, which could potentially cause maladaptation to local environmental conditions. Our study found a positive impact on model fit and accuracy of breeding values from biologically relevant modelling of population structure through contemporary groups implemented in the pedigree. Additionally, the proposed model resulted in changes in genetic correlations between the investigated traits. Such changes affect inference, not only the evolutionary response to selection, but also organismal modularity and phenotypic integration.

## Conclusions

The appropriate modelling of population structure in genetic evaluations is critical for unbiased estimates of genetic parameters. Ignorance or inappropriate modelling of population structure can produce an inferior fit of the models and lower accuracy of genetic parameters. Our study found a positive impact on model fit and accuracy of breeding values from biologically sensible modelling of population structure through contemporary groups implemented in the pedigree. The appropriate modelling of population structure was found especially critical for traits involved in local adaptation. This approach is suitable especially for wind-pollinated species with extensive long-distance pollen flow. Additionally, the proposed model resulted in changes in genetic correlations between the investigated traits. Such changes can affect inference, not only about the evolutionary response to selection but about organismal modularity and phenotypic integration.

## Methods

Coastal Douglas-fir was introduced in New Zealand during the 1950s through the establishment of a provenance test covering genetic material from Washington, Oregon, and limited representation from California [46]. The results of the early evaluation showed superior growth performance of provenances from Oregon and California, therefore, the selection of new breeding material was focused on these geographical areas. A collection of seed from trees at the original wild stands was imported to New Zealand, and a new provenance/progeny test was established in 1996 [47]. The material used to plant at two New Zealand environments (Kaingaroa, latitude 38^∘^ 17’ S, and Gowan Hill, latitude 45^∘^ 52’ S) in 1996 were collected from populations in two US regions (California and Oregon), ranging in latitude from 36^∘^ to 48^∘^ N along the western coast of the USA. Each experiment includes 30 replications of 7 sets. Each set contains 34 open-pollinated families and 2 controls. The provenances were represented equally within each of the set. A detailed description of the material is provided in a previous study [42]. Tree diameter at breast height (DBH) was measured at ages 11 (DBH1 [mm]) and 21 years (DBH2 [mm]). Trees were also assessed for straightness (STR1 and STR2) and malformation (MAL1 and MAL2) at the same ages. Straightness was scored on a scale of 1 to 9 [48] where one represented a crooked stem and nine a straight stem. Similarly, malformation was scored on scale of 1 to 9 where: 1 - tree with multiple leaders, 2 - tree with two leaders, 3 - main stem shifted for more than half of its diameter, 4 - main stem shifted for less than half of its diameter, 5 - tree with multiple ramicorns instead of main leader, 6 - tree has three and more distinctive ramicorns, 7 - tree has two distinctive ramicorns, 8 - tree has one distinctive ramicorn, 9 - tree is clear of any stem shift, multiple leaders or ramicorns. Acoustic wave velocity (VEL1 [km/s]), as an indirect measure of wood stiffness, was measured by HITMAN ST300 (Fibre-gen, Christchurch, New Zealand) at age 11 years. Needle retention describes the proportion of needles retained (NR2 - measured only at Kaingaroa and scored on a scale of 1 - bad to 6 - good, reflecting the damage of needles of different ages) was measured at age 21 years. Branching pattern (BR2) was scored on 9 degrees scale where: 1 - one whorl per year with one set of branches, 2 - one whorl per year with two sets of branches, 3 - one whorl per year with multiple sets of branches, 4 - short internode with multiple sets of branches per whorl, 6 - 9 multinodal trees with increasing score as frequency of nodes increases. Acceptability (AC2) was scored as a binary trait at age 21 years. All class variables (STR, MAL, BR, NR) were transformed into the normal score [49]. Variance components and heritabilities for the investigated traits were estimated using a mixed linear model implemented in the ASReml-R statistical package [50] as follows:

$$\boldsymbol{y}= \boldsymbol{X}\boldsymbol{\beta}+\boldsymbol{Zg}+\boldsymbol{Zr}+\boldsymbol{Zr(s)}+\boldsymbol{e} $$

where ***y*** is the vector of measurements, ***β*** is the vector of fixed effects such as intercept and control, ***g*** is the vector of random individual tree additive genetic effects following var(***g***) ∼N(0,***A***$\sigma _{g}^{2}$), where ***A*** is the average numerator relationship matrix [51] and $\sigma _{g}^{2}$ is additive genetic variance, ***r*** is the vector of random replication effects following var(***r***) ∼N(0,***I***$\sigma _{r}^{2}$), where $\sigma _{r}^{2}$ is replication variance and ***I*** is identity matrix, ***r(s)*** is the vector of random set nested within replication effects following var(***r(s)***) ∼N(0,***I***$\sigma _{r(s)}^{2}$), where $\sigma _{r(s)}^{2}$ is set nested within replication variance, ***e*** is the vector of random residuals following var(***e***) ∼N(0,***I***$\sigma _{e}^{2}$), where $\sigma _{e}^{2}$ is residual variance, and ***X*** and ***Z*** are the incidence matrices assigning the effects from fixed and random vectors to measurements in vector ***y***. Additionally, the provenance term was investigated through three alternative scenarios: 1) used as a fixed term (ABLUP-F), and considered as the default model; 2) implemented as contemporary genetic groups directly in the pedigree and modeling paternal contribution coming from an independent genetic group (phantom group) common across all provenances (ABLUP-CG1); 3) implemented as contemporary genetic groups directly in the pedigree, modeling both maternal and paternal origin from the same genetic group (ABLUP-CG2) [52]. In addition, the model with provenance as a random term was used to test for genetic divergence in quantitative traits (Q _ST_) (ABLUP-R) [53]. Genetic correlations between traits within site and between traits across sites were estimated using bivariate mixed linear model implemented in the ASReml-R statistical package [50] as follows:

$$\boldsymbol{Y}=\boldsymbol{X}\boldsymbol{\beta}+\boldsymbol{Zg}+\boldsymbol{Zr}+\boldsymbol{Zr(s)}+\boldsymbol{e} $$

where ***Y*** is the matrix of measurements, ***g*** is the random vector of individual tree additive genetic effects following var(***g***) ∼N(0,G1), where G1 is the additive genetic variance-covariance structure following G1= $\left [\begin {array}{ll} \sigma _{g_{1}}^{2} & \sigma _{g_{1}g_{2}} \\ \sigma _{g_{2}g_{1}} & \sigma _{g_{2}}^{2}\\ \end {array}\right ]\bigotimes $***A***, where $\sigma _{g_{1}}^{2}$ and $\sigma _{g_{2}}^{2}$ are the additive genetic variances for the 1^st^ and 2^nd^ trait, $\sigma _{g_{1}g_{2}}$ and $\sigma _{g_{2}g_{1}}$ are the additive genetic covariances between the 1^st^ and 2^nd^ trait, and $\bigotimes $ is the Kronecker product, ***r*** is the random vector of replication effects following var(***r***) ∼N(0,G2), where G2 is the replication variance-covariance structure following G2= $\left [\begin {array}{ll}\sigma _{r_{1}}^{2} & 0 \\ 0 & \sigma _{r_{2}}^{2}\\ \end {array}\right ]\bigotimes $***I***, where $\sigma _{r_{1}}^{2}$ and $\sigma _{r_{2}}^{2}$ are the replication variances for the 1^st^ and 2^nd^ trait, ***r(s)*** is the random vector of the set nested within replication effects following var(***r(s)***) ∼N(0,G3), where G3 is the set nested within replication variance-covariance structure following G3= $\left [\begin {array}{ll}\sigma _{r(s)_{1}}^{2} & 0 \\0 & \sigma _{r(s)_{2}}^{2}\\ \end {array}\right ]\bigotimes $***I***, where $\sigma _{r(s)_{1}}^{2}$ and $\sigma _{r(s)_{2}}^{2}$ are set nested within replication variances for the 1^st^ and 2^nd^ trait, ***e*** is the random vector of residual effects following var(***e***) ∼N(0,R), where R is the residual variance-covariance structure following R=$\left [\begin {array}{ll}\sigma _{e_{1}}^{2} & \sigma _{e_{1}e_{2}} \\ \sigma _{e_{2}e_{1}} & \sigma _{e_{2}}^{2}\\ \end {array}\right ]\bigotimes $***I***, where $\sigma _{e_{1}}^{2}$ and $\sigma _{e_{2}}^{2}$ are the residual variances for the 1^st^ and 2^nd^ trait, $\sigma _{e_{1}e_{2}}$ and $\sigma _{e_{2}e_{1}}$ are the residual covariances between the 1^st^ and 2^nd^ trait. The narrow sense heritabilities for traits following normal distribution were estimated as follows:

$$\widehat{h}^{2}=\frac{\widehat{\sigma}_{g}^{2}}{\widehat{\sigma}_{g}^{2}+\widehat{\sigma}_{e}^{2}} $$

The narrow-sense heritability for binary traits was estimated as follows:

$$\widehat{h}^{2}=\frac{\widehat{\sigma}_{g}^{2}}{\widehat{\sigma}_{g}^{2}+\theta\frac{\pi^{2}}{3}} $$

where *θ* is the over/under dispersion coefficient and *π* is 3.14159.

The genetic divergence in quantitative traits was estimated as follows:

$$Q_{ST}=\frac{\widehat{\sigma}_{p}^{2}}{\widehat{\sigma}_{p}^{2}+2\widehat{\sigma}_{g}^{2}} $$

where $\sigma _{p}^{2}$ is the provenance variance. The genetic correlations were estimated as follows:

$$r_{G}=\frac{\widehat{\sigma}_{g_{i}g_{j}}}{\sqrt{\widehat{\sigma}_{g_{i}}^{2}\widehat{\sigma}_{g_{j}}^{2}}} $$

where $\sigma _{g_{i}g_{j}}$ is the additive genetic covariance between the j^th^ and i^th^ trait, and $\sigma _{g_{i}}^{2}$ and $\sigma _{g_{j}}^{2}$ are the additive genetic variances for the i^th^ and j^th^ trait. The standard errors for variance components and genetic parameters were estimated by using Taylor series approximation. Agreement between the correlation matrices obtained from the investigated model was tested through a Mantel test by using the ’MantelCor’ function implemented in the ’evolqg’ R package [54]. Modularity of the investigated traits was tested through a community detection algorithm [55] by using ’LModularity’ function implemented in the ’evolqg’ R package [54]. Breeding value accuracy was estimated as follows:

$$r=\sqrt{1-\frac{PEV}{\widehat{\sigma}_{g}^{2}}} $$

where PEV is the prediction error variance [56], estimated as the square of standard errors for breeding value estimates.

## Supplementary information

**Additional file 1** MS Excel workbook containing all data used in the analyses

## Data Availability

All data used in the study are available in Additional file 1.

## Abbreviations

ABLUP-F: Pedigree-based individual tree model using provenance as fixed term
ABLUP-GC1: Pedigree-based individual tree model implementing provenance as contemporary groups directly in pedigree and assuming both maternal and paternal contribution from the same genetic group
ABLUP-GC2: Pedigree-based individual tree model implementing provenance as contemporary groups directly in pedigree and assuming paternal contribution originated from the phantom genetic group unrelated to maternal provenances
ABLUP-R: Pedigree-based individual tree model using provenance as random term
AC2: Acceptability scored at age of 21 years
AIC: Akaike’s Information Criterion
BR2: Branching pattern measured at age of 21 years
DBH1: Diameter at breast height measured at age of 11 years
DBH2: Diameter at breast height measured at age of 21 years
F _ST_: Fixation index - population differentiation due to genetic structure
G _ST_: Population genetic differentiation (an extension of F _ST_ for multi-allelic markers)
GxE: Genotype by environment interaction
MAL1: Stem malformation scored at age of 11 years
MAL2: Stem malformation scored at age of 21 years
N: North
NR2: Needle retention scored at age of 21 years
PEV: Prediction error variance
Q _ST_: Genetic differentiation among populations expressed by quantitative trait
S: South
SNC: Swiss needle cast
STR1: Stem straightness scored at age of 11 years
STR2: Stem straightness scored at age of 21 years
USA: The United States of America
VEL1: Acoustic wave velocity measured at age of 11 years
